# DBC1 (Deleted in Breast Cancer 1) modulates the stability and function of the nuclear receptor Rev-erbα

**DOI:** 10.1042/BJ20121085

**Published:** 2013-04-12

**Authors:** Claudia C. S. Chini, Carlos Escande, Veronica Nin, Eduardo N. Chini

**Affiliations:** Laboratory of Signal Transduction, Department of Anesthesiology, Mayo Clinic College of Medicine, Rochester, MN 55905, U.S.A.

**Keywords:** BMAL1, circadian, Deleted in Breast Cancer 1 (DBC1), protein stability, Rev-erbα, Alas1, δ-aminolaevulinic acid synthase 1, AR, androgen receptor, DBC1, Deleted in Breast Cancer 1, DMEM, Dulbecco's modified Eagle's medium, ER, oestrogen receptor, FBS, fetal bovine serum, FL, full-length, GAPDH, glyceraldehyde-3-phosphate dehydrogenase, GSK3β, glycogen synthase kinase 3β, HA, haemagglutinin, HDAC, histone deacetylase, HEK, human embryonic kidney, HFD, high-fat diet, KO, knockout, LZ, leucine zipper, MEF, mouse embryonic fibroblast, NCoR, nuclear receptor co-repressor, PGC-1α, peroxisome-proliferator-activated receptor γ co-activator 1α, siRNA, small interfering RNA, WT, wild-type

## Abstract

The nuclear receptor Rev-erbα has been implicated as a major regulator of the circadian clock and integrates circadian rhythm and metabolism. Rev-erbα controls circadian oscillations of several clock genes and Rev-erbα protein degradation is important for maintenance of the circadian oscillations and also for adipocyte differentiation. Elucidating the mechanisms that regulate Rev-erbα stability is essential for our understanding of these processes. In the present paper, we report that the protein DBC1 (Deleted in Breast Cancer 1) is a novel regulator of Rev-erbα. Rev-erbα and DBC1 interact in cells and *in vivo*, and DBC1 modulates the Rev-erbα repressor function. Depletion of DBC1 by siRNA (small interfering RNA) in cells or in DBC1-KO (knockout) mice produced a marked decrease in Rev-erbα protein levels, but not in mRNA levels. In contrast, DBC1 overexpression significantly enhanced Rev-erbα protein stability by preventing its ubiquitination and degradation. The regulation of Rev-erbα protein levels and function by DBC1 depends on both the N-terminal and C-terminal domains of DBC1. More importantly, in cells depleted of DBC1, there was a dramatic decrease in circadian oscillations of both Rev-erbα and BMAL1. In summary, our data identify DBC1 as an important regulator of the circadian receptor Rev-erbα and proposes that Rev-erbα could be involved in mediating some of the physiological effects of DBC1.

## INTRODUCTION

The circadian clock generates oscillations in many physiological processes and behaviour, and allows the organism to adapt to daily changes in environment. At the molecular level, the cellular rhythms are generated and maintained through regulation of clock proteins such as BMAL1, CLOCK, PERIOD and Cryptochrome (CRY) [1,2]. Circadian clock gene expression is altered in human diseases, and mutations in clock genes disrupt diverse physiological processes such as response to genotoxic stress, cell-cycle regulation, metabolism and aging [1,3].

The nuclear receptor Rev-erbα (NR1D1; nuclear receptor subfamily 1, group D, member 1) has been shown to be a major regulator of circadian rhythm, metabolism and adipogenesis. Rev-erbα represses transcription of several genes that control these cellular processes. For instance, Rev-erbα is part of the core clock machinery and represses the expression of the transcription factors BMAL1 and CLOCK [4,5], key components of the mammalian circadian clock, and regulators of the circadian genes *PERIOD* and *CRY* [1]. In addition, Rev-erbα shows a strong circadian pattern in many tissues, and is important in the circadian control of metabolism [2]. The metabolic functions of Rev-erbα involve regulation of glucose homoeostasis and energy metabolism through repression of gene expression of several gluconeogenic genes such as *PEPCK* (phosphoenolpyruvate carboxykinase), *G6Pase* (glucose 6-phosphatase) [6] and the metabolic transcriptional regulator *PGC-1α* (peroxisome-proliferator-activated receptor γ co-activator 1α; also known as *PPARGC1A*) [7]. During adipogenesis Rev-erbα has a complex role, since its expression is induced during the early stages of adipogenesis, but its degradation is required for continued adipocyte differentiation [8].

Rev-erbα transcription repressor activity depends on a complex formed by the NCoR (nuclear receptor co-repressor) and HDAC (histone deacetylase) 3 [4]. NCoR binds to and activates the deacetylase activity of HDAC3 [9], and both proteins are required for the binding of Rev-erbα to target promoters and its repression activity [4,10]. Rev-erbα was considered an orphan receptor for many years, until the prosthetic group haem was identified as the ligand for Rev-erbα [6,11]. Binding of haem to Rev-erbα stimulates its interaction with the NCoR–HDAC3 complex and enhances repression of Rev-erbα target genes [6,11]. It has also been shown that Rev-erbα tightly regulates the synthesis of its own ligand [7]. Cellular haem levels are controlled by the enzyme Alas1 (δ-aminolaevulinic acid synthase 1), the rate-limiting enzyme in haem synthesis. Binding of haem to Rev-erbα represses PGC-1α, a potent inducer of Alas1 and haem synthesis, whereas depletion of Rev-erbα derepress PGC-1α, resulting in an increase in haem levels [7].

Another mechanism of regulation of Rev-erbα is phosphorylation by GSK3β (glycogen synthase kinase 3β) [12]. GSK3β phosphorylates Rev-erbα and prevents its rapid proteasomal degradation. Lithium, an inhibitor of GSK3β, leads to degradation of Rev-erbα and activation of the clock gene *BMAL1* [12]. This phosphorylation and stabilization of Rev-erbα protein levels has an important role in Rev-erbα functions, since mutations of the GSK3β phosphorylation sites that make the protein resistant to degradation interfere with processes such as adipogenesis and oscillations of circadian genes [8,12]. Owing to the importance of regulating cellular Rev-erbα levels for many physiological processes, it is essential to understand the molecular pathways that control Rev-erbα stability and function.

The nuclear protein DBC1 (Deleted in Breast Cancer 1) has been shown previously to be a co-activator for some nuclear receptors such as ER (oestrogen receptor) α and β and the AR (androgen receptor) [13–16]. DBC1 binds to these receptors and modulates their transcriptional activity. Besides modulating transcriptional activity, we [17,18] and others [19,20] have shown that DBC1 regulate the deacetylases HDAC3 and SIRT1. DBC1 binds to both deacetylases and inhibits their deacetylase activity, regulating their functions. Moreover, we found that DBC1 regulates lipid accumulation, and that DBC1-deficient mice are protected from HFD (high-fat diet)-induced liver steatosis and inflammation [18], indicating a role for DBC1 in metabolism.

In view of the importance of DBC1 in metabolism, and in HDAC3 and nuclear receptor regulation, we investigated whether DBC1 regulates the transcriptional repressor Rev-erbα. Our data reveal that DBC1 binds to Rev-erbα, and modulates its transcriptional activity through stabilization of Rev-erbα protein levels. In addition, DBC1 regulates the circadian expression of Rev-erbα and BMAL1. In summary, the results of the present study identified DBC1 as a new regulator of the Rev-erbα receptor and suggests that DBC1 may be a modulator of the circadian and metabolic functions of Rev-erbα.

## EXPERIMENTAL

### Cell culture

HEK (human embryonic kidney)-293T cells, MEFs (mouse embryonic fibroblasts) and NIH 3T3 cells were maintained in high-glucose DMEM (Dulbecco's modified Eagle's medium) supplemented with 10% FBS (fetal bovine serum), 100 units/ml penicillin and 100 mg/ml streptomycin (Invitrogen). INS-1 cells were cultured as described previously [18].

### Reagents and antibodies

Except when specified, all reagents and chemicals were purchased from Sigma Chemicals. The anti-Rev-erbα antibodies were from Cell Signaling Technology and Abcam. Phospho-Rev-erbα (Ser^55^/Ser^59^) antibody and TSA (trichostatin A) were from Cell Signaling Technology. Antibodies against SIRT1, HDAC3 and HA (haemagglutinin) were from Abcam and the anti-DBC1 antibody was from Bethyl Laboratories. The proteasome inhibitor carbobenzoxy-L-leucyl-L-leucyl-leucinal (MG-132) was from Enzo Life Sciences and GSK4112 was from Calbiochem.

### Plasmids and transfections

pcDNA3.1-FLAG–hRev-erbα was generously provided by Dr Mitchell Lazar (University of Pennsylvania, PA, U.S.A.) and the mouse Bmal1-luciferase vector by Dr Masaaki Ikeda (University of Saitama Medical School, Saitama, Japan). S55D/S59D mutation of FLAG–Rev-erbα (S55D/S59D) was generated by site-directed mutagenesis using the QuikChange® kit (Stratagene). DBC1 and HDAC plasmids have been described previously [16]. All transient transfection assays were performed using Lipofectamine™ 2000 (Invitrogen) according to the manufacturer's instructions. Cells were harvested after 48 h of transfection. For experiments studying the interaction between FLAG–Rev-erbα and HA–DBC1, cells were treated for 6 h with 10 μM MG-132 before harvesting. When the Rev-erbα agonist GSK4112 was added, cells were treated for 16 h with 10 μM GSK4112 in the presence of 2 μM MG-132 in DMEM supplemented with 0.5% FBS.

For repression assays, cells were grown in 24-well plates and transfected with 50 ng of Bmal1-luciferase reporter, 5 ng of pRL-CMV *Renilla* luciferase reporter (Promega), 25–100 ng of FLAG–Rev-erbα and 200–600 ng of HA–DBC1. After 48 h, cells were lysed in passive lysis buffer (Promega) and their luciferase activity was assayed using a dual-luciferase reporter assay kit from Promega. Luciferase units were normalized to *Renilla* expression. Relative luciferase activity was expressed as fold activity over the control group (control vector). Each experiment was performed at least three times in triplicate.

### siRNA (small interfering RNA)

siRNA against DBC1 was synthesized by Dharmacon. The siRNA duplexes were 21 bp as follows: DBC1 siRNA sense strand, 5′-AAACGGAGCCUACUGAACAUU-3′. Non-targeting siRNA (Dharmacon) was used as control. Transfections were performed with 150 nM siRNA using DharmaFECT reagent according to the manufacturer's instruction. Cells were harvested 72 h after transfection.

### Immunoprecipitation and Western blot analysis

Mouse tissues and cultured cells were lysed in buffer containing 20 mM Tris/HCl (pH 8.0), 100 mM NaCl, 1 mM EDTA and 0.5% Nonidet P40, supplemented with 5 mM NaF, 50 mM 2-glycerophosphate and a protease inhibitor cocktail (Roche). All mice in the present study were maintained in the Mayo Clinic animal facility and all experimental protocols were approved by the institutional animal care and use committee at Mayo Clinic (protocol A33209). All studies were performed according to the methods approved in the protocol. After 20 min of lysis, protein lysates were cleared by centrifugation at 12000 ***g*** at 4°C for 10 min. The resulting supernatants were quantified using the Bio-Rad Laboratories protein assay and used as whole-cell lysates or for immunoprecipitation. Immunoprecipitation was performed for 1–2 h at 4°C using 1–2 mg of protein lysates, specific antibodies and Protein A/G–agarose beads (Santa Cruz Biotechnology). Proteins were separated by SDS/PAGE (8.5% gel) and transferred on to Immobilon membranes (Millipore). Membranes were probed with the indicated antibodies, followed by incubation with HRP (horseradish peroxidase)-conjugated anti-mouse or anti-rabbit secondary antibody. Western blots were developed using SuperSignal West Pico Chemiluminescent substrate according to the manufacturer's instructions (Thermo Scientific). Films were scanned and protein bands were quantified by densitometry using ImageJ software. Protein levels were normalized to actin or tubulin levels.

### Serum shock and synchronization study

NIH 3T3 cells were transfected with a control and a DBC1 siRNA oligonucleotide. At 48 h after transfection, cells were starved by incubation in 0.5% FBS-containing medium for 16 h and then synchronized by serum shock. Serum shock treatment involves exposing the cells to 50% horse serum diluted in DMEM for 2 h, washing cells with PBS and replacing medium with 0.5% FBS-containing medium. Cells were collected for both protein analysis and RNA extraction at the indicated times after serum shock. When the experiment was performed in MEFs, confluent cells were arrested before serum shock by leaving them in the same medium for 4 days before serum shock.

### Real-time PCR

Total mRNA was prepared using the RNeasy kit (Qiagen). cDNA was synthesized using the QuantiTect Reverse Transcription kit (Qiagen). Commercially available TaqMan gene expression probes for mouse and human Rev-erbα, BMAL1, GAPDH (glyceraldehyde-3-phosphate dehydrogenase) (endogenous control) and DBC1 were obtained from Applied Biosystems. The quantitative real-time PCR was performed in triplicate according to the manufacturer's instructions. mRNA abundance was evaluated by the standard curve method and the value of Rev-erbα obtained was divided by the GAPDH value to obtain a normalized value. All experiments were performed at least three times.

### Statistical analysis

Data are expressed as means±S.D. from at least three independent experiments and were analysed using unpaired *t* test. The significance was set at *P*<0.05.

## RESULTS

### DBC1 is a Rev-erbα-interacting protein

**Figure 1:**
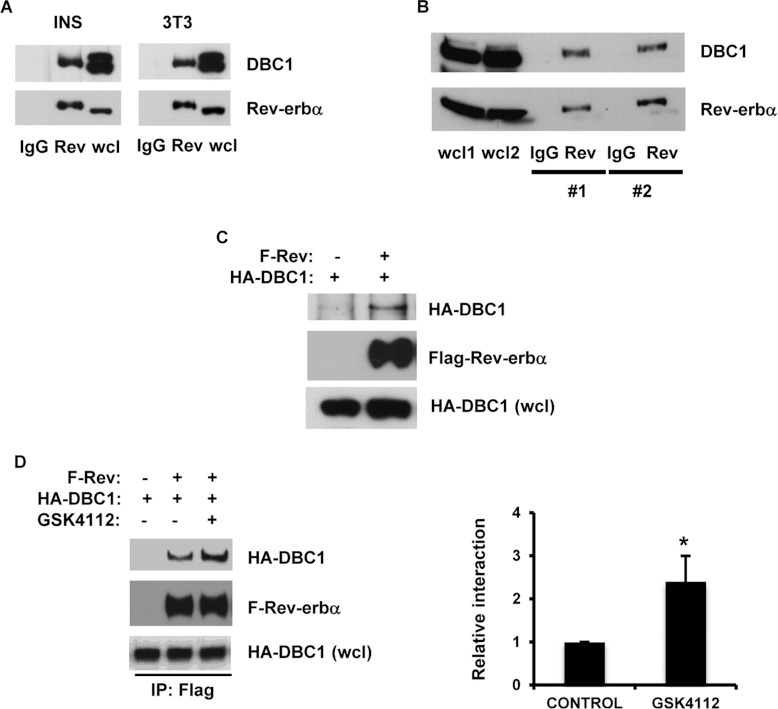
DBC1 interacts with Rev-erbα (**A**) INS-1 and NIH 3T3 cell lysates were immunoprecipitated with anti-Rev-erbα antibody and immunoblotted with anti-DBC1 and anti-Rev-erbα antibodies. (**B**) Pancreas protein homogenates from two different mice (#1 and #2) were immunoprecipited with anti-Rev-erbα antibody and immunoblotted with anti-DBC1 and anti-Rev-erbα antibodies. (**C**) HEK-293T cells were transfected with FLAG–Rev-erbα and HA–DBC1. Cell lysates were immunoprecipitated with an anti-FLAG antibody and immunoblotted with anti-HA and anti-FLAG antibodies. (**D**) Representative experiment where HEK-293T cells were transfected with HA–DBC1 in the presence of FLAG-Rev-erbα or empty vector. At 16 h before lysis, cells were treated with vehicle or the Rev-erbα agonist GSK4112. Rev-erbα was immunoprecipitated with an anti-FLAG antibody and immunoprecipitates and cell lysates were immunoblotted with anti-FLAG and anti-HA antibodies. The histogram shows the increase in the DBC1–Rev-erbα interaction upon addition of GSK4112 in four independent experiments. Results are means±S.D. *P<0.05. IP, immunoprecipitation; wcl, whole-cell lysate.

It has been shown previously that DBC1 binds to and regulates some nuclear receptors such as the ERs and ARs [13–16]. In the present study, we explored whether DBC1 also interacts with the nuclear receptor Rev-erbα. We performed immunoprecipitation of Rev-erbα from cell extracts of NIH 3T3 and INS-1 cells, and also from mice pancreas homogenates. After immunoblotting, we found DBC1 present in the Rev-erbα immunoprecipitates (Figures 1A and 1B), indicating that DBC1 interacts with Rev-erbα in cells and *in vivo*. The interaction between these two proteins was also observed when we transfected HEK-293T cells with FLAG–Rev-erbα and HA–DBC1. FLAG–Rev-erbα was immunoprecipitated with an anti-FLAG antibody and DBC1 was detected in the Rev-erbα immunoprecipitates (Figure 1C). We also investigated whether Rev-erbα agonists regulate the Rev-erbα–DBC1 interaction. Haem was identified as the endogenous ligand for Rev-erbα, but some synthetic ligand agonists such as GSK4112 have been described previously [21–23]. Interestingly, we found that in HEK-293T cells transfected with FLAG–Rev-erbα and HA–DBC1, the addition of GSK4112 further increased the interaction between DBC1 and Rev-erbα (Figure 1D). Together, these data establish that DBC1 and Rev-erbα interact in cells and *in vivo*, and this interaction is regulated by the addition of a Rev-erbα agonist.

### DBC1 regulates Rev-erbα-mediated gene expression

**Figure 2:**
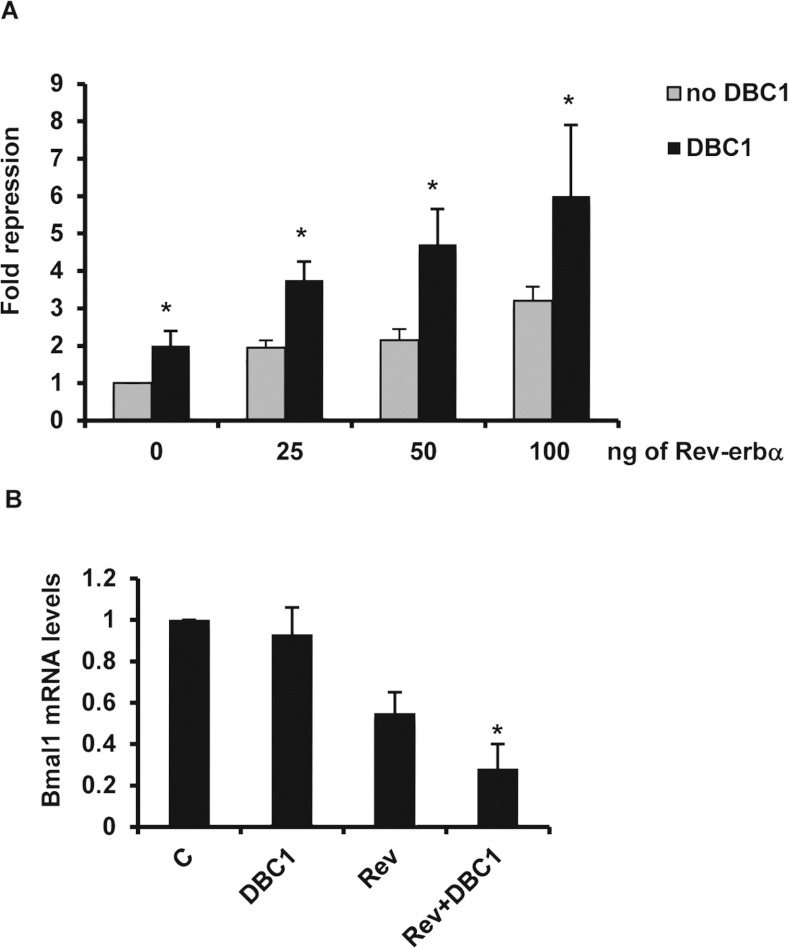
DBC1 regulates Rev-erbα-mediated gene repression (**A**) BMAL1-luciferase reporter activity was measured in HEK-293T cells transfected with different amounts of FLAG–Rev-erbα (25–100 ng) in the presence and absence of HA–DBC1 (400 ng). The histogram shows the means±S.D. for three experiments. (**B**) *BMAL1* mRNA levels in HEK-293T cells transfected with vector (control; C), HA–DBC1 (DBC1), FLAG–Rev-erbα (Rev) or a combination of HA–DBC1 and FLAG–Rev-erbα (Rev+DBC1). The histogram shows the means±S.D. for three experiments. *P<0.05

To assess the functional significance of the DBC1–Rev-erbα interaction, we investigated whether DBC1 regulates the transcription repression activity of Rev-erbα. One of the genes repressed by Rev-erbα is the circadian gene *BMAL1* [4]. Rev-erbα directly binds to the promoter of *BMAL1* and represses *BMAL1* gene expression. Transfection of different amounts of Rev-erbα plasmid in HEK-293T cells with a luciferase reporter gene under the control of the *BAML1* promoter inhibited *BMAL1* expression, confirming that Rev-erbα was repressing *BMAL1* transcription (Figure 2A). We also found that our levels of repression of *BMAL1* transcription by Rev-erbα were similar to the levels described previously [4]. When DBC1 was transfected together with Rev-erbα, it significantly increased the repression mediated by Rev-erbα at all of the Rev-erbα concentrations tested (Figure 2A). The effect of DBC1 on the repression of the BMAL1-luciferase activity was also detected when DBC1 was transfected in the absence of Rev-erbα (Figure 2A), possibly due to an effect of DBC1 in endogenous Rev-erbα.

To confirm the effect of DBC1 in Rev-erbα function, we measured the levels of endogenous *BMAL1* mRNA when we overexpressed DBC1 in cells. When HEK-293T cells were transfected with Rev-erbα, there was a decrease in endogenous *BMAL1* mRNA levels, consistent with an effect of Rev-erbα in repressing *BMAL1* transcription. Transfection of DBC1 together with Rev-erbα further decreased the *BMAL1* mRNA levels (Figure 2B). In contrast, when DBC1 was overexpressed alone, no significant effect was observed in endogenous *BMAL1* mRNA levels. These data indicate that DBC1 regulates the repression function of Rev-erbα.

### DBC1 regulates Rev-erbα protein levels

**Figure 3:**
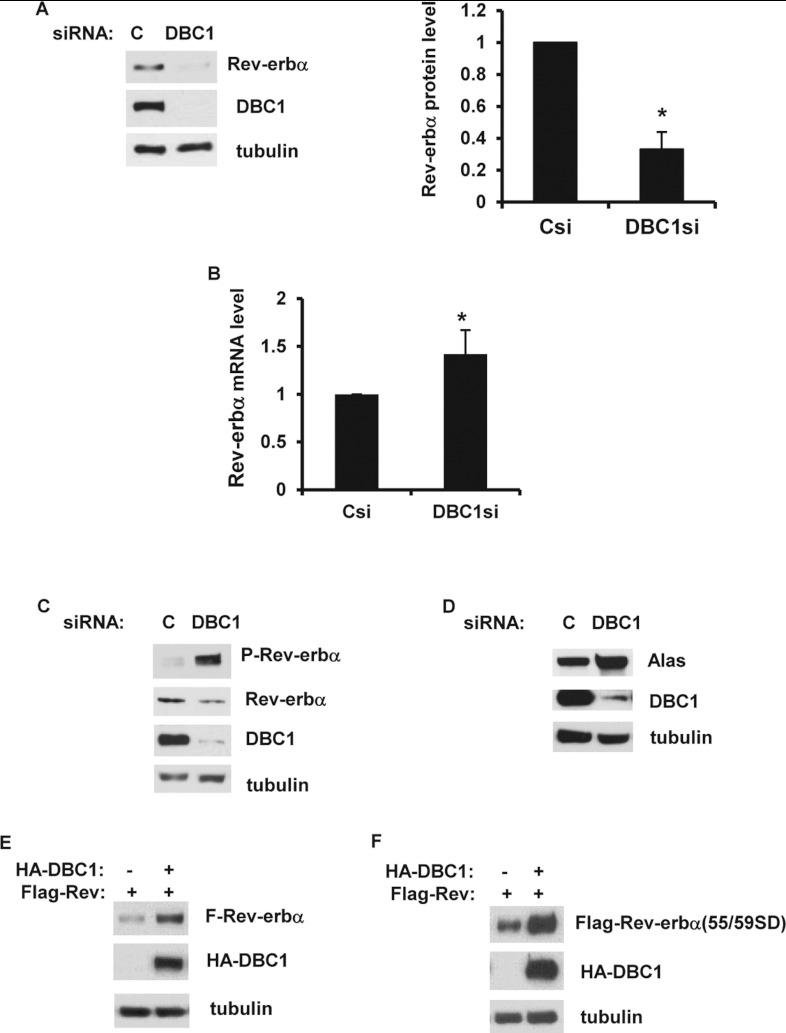
Rev-erbα protein levels, but not mRNA levels, are regulated by DBC1 (**A**) Cell lysates of NIH 3T3 cells treated with control siRNAs (Csi) and DBC1 siRNAs (DBC1si) were analysed by immunoblotting with anti-Rev-erbα, anti-DBC1 and anti-tubulin antibodies. The histogram shows the means±S.D. for five independent experiments. *P<0.05 (**B**) Rev-erbα mRNA levels were measured in NIH 3T3 cells transfected with Csi and DBC1si. mRNA levels were quantified by real-time PCR. The histogram shows means±S.D. for three experiments. *P<0.05 (**C**) NIH 3T3 cells treated with control and DBC1 siRNAs were immunoblotted with anti-Rev-erbα, anti-p-Rev-erbα, anti-DBC1 and anti-tubulin antibodies. (**D**) NIH 3T3 cells treated with control and DBC1 siRNAs were immunoblotted with anti-Alas1, anti-DBC1 and anti-tubulin antibodies. (**E** and **F**) HEK-293T cells were transfected with FLAG–Rev-erbα (**E**) or FLAG–Rev-erbα (S55D/S59D) (**F**) in the presence of HA–DBC1 or empty vector. Cell lysates were immunoblotted with anti-FLAG, anti-HA and anti-tubulin antibodies. C, control.

As a first step to understanding the molecular mechanism by which DBC1 regulates Rev-erbα function, we examined Rev-erbα protein levels under conditions where DBC1 protein levels were decreased. In NIH 3T3 cells treated with siRNA, we observed lower levels of the Rev-erbα protein in DBC1 siRNA-treated cells than in control siRNA cells (Figure 3A). A similar decrease in Rev-erbα levels was also observed in INS-1 cells treated with DBC1 siRNA (Supplementary Figure S1 at http://www.biochemj.org/bj/451/bj4510453add.htm). In addition, in MEFs obtained from DBC1-KO (knockout) mice the steady-state levels of Rev-erbα were remarkably lower than in WT (wild-type) MEFs (Supplementary Figure S2 at http://www.biochemj.org/bj/451/bj4510453add.htm).

We next investigated whether DBC1 regulates Rev-erbα protein levels through modulation of Rev-erbα transcription. However, we found that transfection of DBC1 siRNA did not decrease Rev-erbα mRNA levels (Figure 3B). Also, in DBC1-KO MEFs there was no significant decrease in Rev-erbα mRNA (Supplementary Figure S2). These results indicate that knockdown of DBC1 did not inhibit Rev-erbα gene expression. In fact, there was a small, but significant, increase in Rev-erbα mRNA levels when we knocked down DBC1 by siRNA (Figure 3B).

Interestingly, we noticed that whereas total Rev-erbα protein levels were lower in DBC1 siRNA-treated cells than in control cells, the levels of phospho-Rev-erbα (Ser^55^/Ser^59^) were higher (Figure 3C). Phosphorylation of Rev-erbα in these sites has been shown to be mediated by GSK3β and to stabilize Rev-erbα [12]. The fact that we saw an increase in phosphorylation of Rev-erbα when DBC1 levels were reduced indicates that Rev-erbα phosphorylation was not inhibited in the absence of DBC1. This increase in phosphorylation may be a compensatory mechanism to stabilize Rev-erbα in the absence of DBC1. Additionally, when DBC1 expression was inhibited by siRNA transfection, or depleted in DBC1-KO MEFs there was an increase in the expression of the protein Alas1 (Figure 3D and Supplementary Figure S2). Alas1 expression has been reported to be repressed by Rev-erbα, indicating that the Rev-erbα pathway is indeed compromised in the absence of DBC1.

The effect of DBC1 on Rev-erbα protein levels was also confirmed by overexpression of HA-DBC1 and FLAG–Rev-erbα in HEK-293T cells. Co-expression of these two proteins increased FLAG–Rev-erbα protein levels compared with FLAG–Rev-erbα expression alone (Figure 3E). Again, we found that DBC1 overexpression did not alter the mRNA levels of endogenous or expressed Rev-erbα (Supplementary Figure S3 at http://www.biochemj.org/bj/451/bj4510453add.htm). Moreover, when we expressed the more stable mutant of Rev-erbα (S55D/S59D) together with DBC1, there was further stabilization of this mutant by the presence of DBC1 (Figure 3F), indicating that DBC1 is regulating Rev-erbα through a GSK3β-independent mechanism.

**Figure 4:**
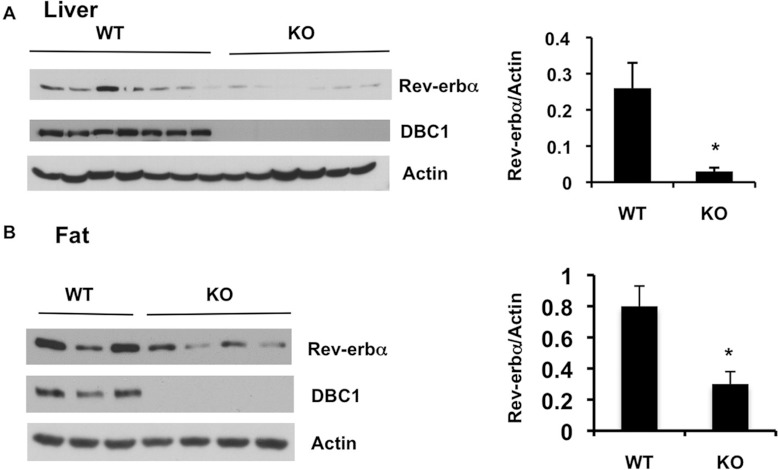
Rev-erbα protein levels are lower in tissues from DBC1-KO mice than control mice (**A**) Liver homogenates from several WT and DBC1-KO mice were immunoblotted with anti-Rev-erbα, anti-DBC1 and anti-actin antibodies. The histogram shows means±S.E.M. (*n*=7 WT; *n*=6 DBC1-KO mice). *P<0.05. (**B**) Fat homogenates from DBC1-KO and WT mice were immunoblotted with anti-Rev-erbα, anti-DBC1 and anti-actin antibodies. The histogram shows means±S.E.M. (*n*=5 WT; *n*=6 DBC1-KO mice). *P<0.05.

To confirm the effects of DBC1 on Rev-erbα *in vivo*, we measured Rev-erbα protein levels in tissue homogenates obtained from DBC1-KO mice. Rev-erbα protein levels were measured by immunoblotting in liver and fat homogenates. Again, we detected significantly lower levels of the Rev-erbα protein in tissues from DBC1-KO mice than in tissues from WT mice (Figures 4A and 4B). Together, these data indicate that DBC1 is required to maintain Rev-erbα protein levels and that the mechanism involved is not regulation of Rev-erbα gene expression.

### DBC1 regulates Rev-erbα protein stability

**Figure 5:**
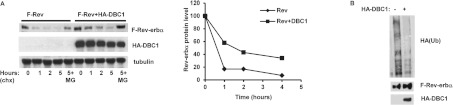
DBC1 regulates Rev-erbα protein stability (**A**) HEK-293T cells were transfected with FLAG–Rev-erbα and empty vector or HA–DBC1. Before lysis cells were treated for different time periods with 100 μg/ml cycloheximide (chx) and for 6 h with 10 μM MG-132 (MG). Cell lysates were immunoblotted with anti-FLAG, anti-HA and anti-tubulin antibodies. The graph shows quantification of Rev-erbα protein levels in the immunoblots relative to tubulin levels. (**B**) HEK-293T cells were transfected with FLAG–Rev-erbα and HA–ubiquitin (Ub) in the presence of empty vector or HA–DBC1. Cell lysates were immunoprecipitated with an anti-FLAG antibody and immunoblotted with anti-HA and anti-FLAG antibodies.

Having shown that DBC1 regulates Rev-erbα protein levels, but not mRNA levels, we next examined whether DBC1 was regulating Rev-erbα protein stability. To test this hypothesis, we determined the effect of DBC1 overexpression on the protein half-life of Rev-erbα. FLAG–Rev-erbα has a short half-life of approximately 30 min, but co-expression with DBC1 markedly increased Rev-erbα protein stability and half-life to approximately 1.5 h (Figure 5A).

In order to determine the molecular mechanism by which DBC1 regulates Rev-erbα stability, we explored the possibility that DBC1 was interacting with Rev-erbα and preventing its degradation. It has been shown previously that Rev-erbα protein levels are regulated by ubiquitination and proteasome degradation [24]. When we expressed FLAG–Rev-erbα with HA–ubiquitin in HEK-293T cells, we observed higher levels of ubiquitinated Rev-erbα when it was expressed alone than when it was co-expressed with DBC1 (Figure 5B), implying that DBC1 is likely to be stabilizing Rev-erbα by preventing its ubiquitination and subsequent degradation.

### DBC1 N-terminal and C-terminal domains are required for the regulation of Rev-erbα

**Figure 6:**
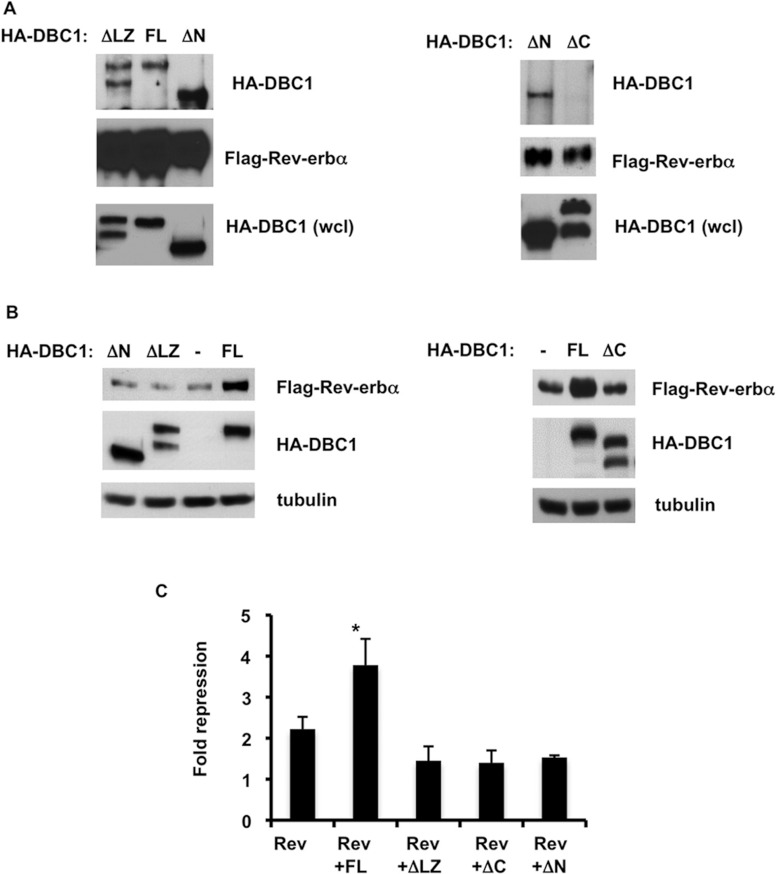
DBC1 N-terminal and C-terminal domains are required for the regulation of Rev-erbα (**A**) HEK-293T cells were transfected with FLAG–Rev-erbα and FL DBC1 or the ΔLZ and ΔN deletions of HA–DBC1 (left-hand panel). In the right-hand panel, cells were transfected with FLAG–Rev-erbα and the ΔN and ΔC deletions of HA–DBC1. Cell lysates were immunoprecipitated with an anti-FLAG antibody and immunoblotted with anti-HA and anti-FLAG antibodies. (**B**) HEK-293T cells were transfected with FLAG–Rev-erbα and FL DBC1 or the ΔLZ and ΔN deletions of HA–DBC1 (left-hand panel). In the right-hand panel, cells were transfected with FLAG–Rev-erbα and FL HA–DBC1 or the ΔC deletion of DBC1. Cell lysates were immunoblotted with anti-FLAG, anti-HA and anti-tubulin antibodies. (**C**) BMAL1-luciferase reporter activity was measured in HEK-293T cells transfected with FLAG–Rev-erbα (50 ng), FL HA–DBC1 (400 ng), ΔLZ (600 ng), ΔN (300 ng) and ΔC (400 ng) deletions of DBC1. The histogram shows means±S.D. for three experiments. *P<0.05.

The interaction between DBC1 and proteins such as nuclear receptors and the deacetylases SIRT1 and HDAC3 occur through the N-terminal region of DBC1 and it mostly depends on its LZ (leucine zipper) domain [13,15,17,18]. To map the region on DBC1 that binds to Rev-erbα, we first tested whether this interaction was dependent on the N-terminal domain of DBC1. In HEK-293T cells transfected with FLAG–Rev-erbα and the N-terminal deletion mutant of DBC1 (Δ1–264) or the LZ deletion mutant of DBC1, we still observed the interaction between DBC1 and Rev-erbα, similar to FL (full-length) DBC1 (Figure 6A). Instead, it was the deletion of the C-terminal domain of DBC1 (Δ704–923) that prevented the association between DBC1 and Rev-erbα. This indicates that, different from other nuclear receptors, Rev-erbα interacts with the C-terminal domain of DBC1 (Figure 6A).

To determine which domains of DBC1 are important for the regulation of Rev-erbα stability, we transfected Rev-erbα alone and in the presence of FL DBC1, ΔN-terminal domain, ΔLZ and ΔC-terminal domain, and measured the steady-state levels of Rev-erbα protein. Although the N-terminal domain of DBC1 is not essential for the association between DBC1 and Rev-erbα, we found that this region is required for stabilization of Rev-erbα. Furthermore, both the C-terminal deletion and the LZ deletion mutants did not stabilize Rev-erbα (Figure 6B), indicating that multiple regions of DBC1 are required for regulation of Rev-erbα stability.

To confirm the importance of the different DBC1 domains in Rev-erbα function, we tested the effect of the expression of the DBC1 mutants on *BMAL1* gene expression. Unlike FL DBC1, all mutants failed to increase repression of BMAL1 transcription by Rev-erbα (Figure 6C). These results support the hypothesis that multiple regions of DBC1 are required for the DBC1 effect on Rev-erbα stability and function. It is possible that the N-terminal and C-terminal regions of DBC1 have different roles in Rev-erbα regulation. Whereas the C-terminus of DBC1 is the region that interacts with Rev-erbα, the N-terminus may bring additional proteins to Rev-erbα which may be necessary to control its stability and function.

### DBC1 regulates the circadian expression of Rev-erbα in cells after serum shock

Rev-erbα is a key regulator of the circadian clock. Rev-erbα expression is regulated in a circadian manner that is controlled both transcriptionally and post-transcriptionally. Furthermore, expression of several circadian genes is dependent on Rev-erbα expression [25,26]. Since DBC1 modulates Rev-erbα stability and function, we explored whether DBC1 is involved in the regulation of cellular circadian rhythm.

**Figure 7:**
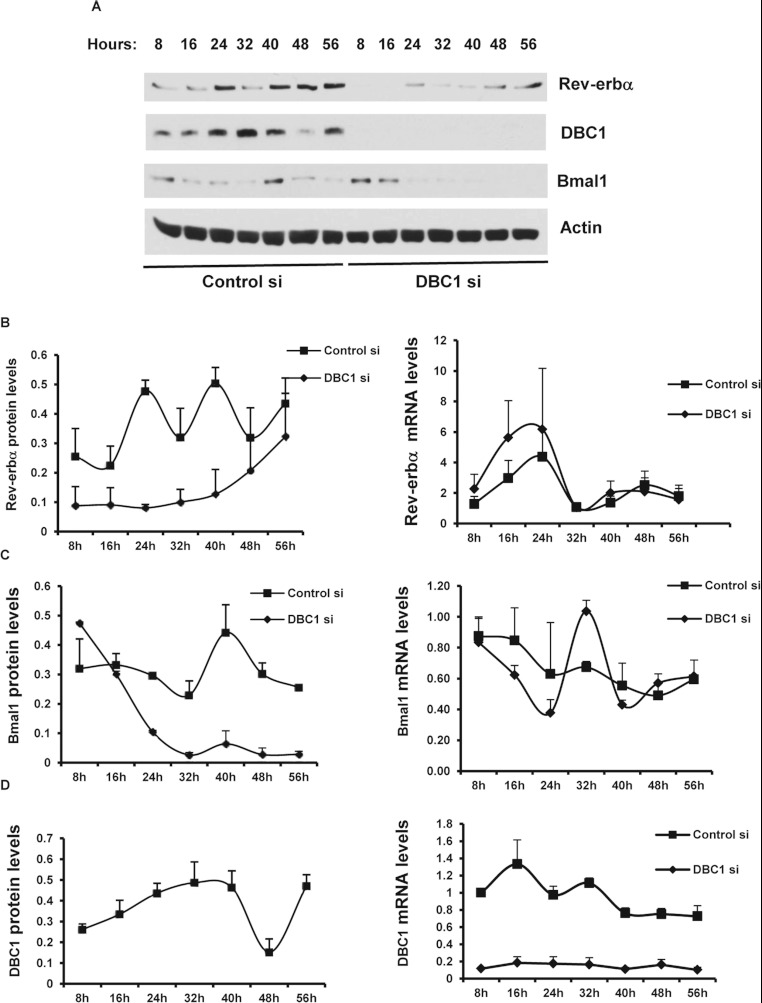
DBC1 regulates circadian expression of Rev-erbα NIH 3T3 cells were transfected with control and DBC1 siRNAs for 60 h, synchronized with serum shock, and then collected at the indicated times for protein and mRNA analysis. (**A**) Representative experiment showing protein levels of Rev-erbα, DBC1, BMAL1 and actin at the indicated times after serum shock. (**B**) Graphs show means±S.E.M. (*n*=3) of Rev-erbα protein (left-hand panel) and mRNA (right-hand panel) levels in control and DBC1 siRNA-transfected cells. (**C**) Graphs show means±S.E.M. (*n*=3) of BMAL1 protein (left-hand panel) and mRNA (right-hand panel) levels in control and DBC1 siRNA-transfected cells. (**D**) Graphs show means±S.E.M. (*n*=3) of DBC1 protein levels in control siRNA-treated cells (left-hand panel) and mRNA levels in control and DBC1 siRNA-transfected cells (right-hand panel) collected at the indicated times after serum shock.

Studies using *in vitro* models have yielded evidence to indicate that peripheral cells are capable of expressing circadian genes oscillations independent of the 12 h light/12 h dark cycle. For example, NIH 3T3 fibroblasts exposed to serum shock show rhythmic fluctuations in mRNA abundance of circadian genes that is modulated by the Rev-erbα protein [12,27]. To study the intrinsic cellular circadian regulation, we performed serum shock in NIH 3T3 cells transfected with control and DBC1 siRNA. We found that Rev-erbα, BMAL1 and DBC1 showed circadian oscillations at both the protein and mRNA level in cells transfected with control siRNA (Figure 7). In contrast, in cells transfected with DBC1 siRNA, there was a dramatic inhibition of the serum shock-induced circadian oscillations of Rev-erbα and BMAL1 proteins compared with cells transfected with control siRNA (Figures 7A–7C). In the case of Rev-erbα, the oscillations in mRNA were similar between control and DBC1 siRNA-transfected cells, with the mRNA levels of Rev-erbα being actually higher at 16 h and 24 h in DBC1 siRNA-transfected cells than in control siRNA-transfected cells (Figure 7B). These results confirm our hypothesis that DBC1 regulates Rev-erbα protein stability and not gene expression.

In the case of BMAL1, we observed higher circadian oscillation in *BMAL1* mRNA levels in DBC1 siRNA-transfected cells than in control siRNA cells. However, at the protein level, the BMAL1 oscillations were lost in the absence of DBC1 (Figure 7C). It is possible that DBC1 regulates BMAL1 in multiple ways. The lower levels of BMAL1 protein during the circadian cycle in the absence of DBC1 suggests that DBC1 regulates other proteins beside Rev-erbα that are responsible for maintaining BMAL1 stability. In fact, it has been reported that BMAL1 expression is regulated by the deacetylase SIRT1 [28,29]. When we measured SIRT1 activity during the circadian cycle, we observed that in DBC1-KO MEFs, SIRT1 activity is very high and did not oscillate like in WT MEFs (Supplementary Figure S4 at http://www.biochemj.org/bj/451/bj4510453add.htm). Thus it is possible that this high activity of SIRT1 in the absence of DBC1 contributes to the final effect in BMAL1 expression.

Furthermore, although DBC1 and Rev-erbα showed circadian oscillations at the protein level, the pattern of oscillation is different between both proteins (Figures 7A, 7B and 7D). It is possible that DBC1 oscillations do not determine the Rev-erbα oscillations, but that the presence of DBC1 is required for the oscillations to happen. Together, these results suggest that DBC1 is a novel regulator of Rev-erbα and the circadian pathways in cells.

## DISCUSSION

Dynamic expression of Rev-erbα is important for several physiological processes similar to the circadian cycle [24,25], adipocyte differentiation [8,22] and regulation of liver fat metabolism [6,25,30]. Defining the mechanisms that control Rev-erbα protein levels and turnover is essential for our understanding of these processes and for the development of therapies. Our study reveals a new pathway involved in Rev-erbα regulation and proposes that DBC1 is an important modulator of the Rev-erbα functions. DBC1 controls Rev-erbα by a mechanism that involves an increase in Rev-erbα protein stability. By preventing Rev-erbα ubiquitination, DBC1 promotes stabilization of Rev-erbα levels and an increase in repression activity of Rev-erbα.

The only mechanism described to date for regulation of Rev-erbα protein stability is phosphorylation by the protein kinase GSK3β [12]. GSK3β controls circadian rhythm in many organisms and phosphorylates many clock proteins [3]. GSK3β phosphorylates Rev-erbα on Ser^55^ and Ser^59^ and this phosphorylation prevents Rev-erbα proteasomal degradation, stabilizing Rev-erbα protein levels [12]. A form of Rev-erbα that is stable and insensitive to the GSK3β inhibitor lithium interferes with expression of circadian genes [12], indicating that GSK3β-dependent phosphorylation of Rev-erbα is important for regulation of the peripheral clock. This degradation pathway involves the E3 ligases Arf-bp1 and Pam, which are required for efficient ubiquitination and degradation of Rev-erbα [24]. Because DBC1 prevents Rev-erbα ubiquitination, it is possible that DBC1 may interfere with the interaction between Rev-erbα and these ubiquitin ligases. However, the effect of DBC1 appears to be independent of GSK3β phosphorylation, since depletion of DBC1 does not prevent phosphorylation of Rev-erbα, and DBC1 can still stabilize a form of Rev-erbα (S55D/S59D) that mimics the phosphorylated state. This suggests that DBC1 regulates a novel pathway that controls Rev-erbα stability.

In addition to Rev-erbα, other nuclear receptors are also regulated by DBC1. However, DBC1 appears to have a complex role in nuclear receptor regulation. In the case of the AR, DBC1 functions as a co-activator and dramatically enhances AR DNA binding and facilitates AR transcriptional activation. In addition, binding of DBC1 to AR is ligand-dependent, involves the N-terminal region of DBC1 and does not significantly affect AR stability [13]. ERα also binds to the N-terminal domain of DBC1, but there are contradictory data on whether DBC1 regulates ERα protein stability [15,16]. Whereas in an earlier report DBC1 was shown to regulate the steady-state level of unliganded protein [15], a more recent study found that DBC1 did not affect the levels of this receptor and that it binds to ERα both in the presence and absence of ligand [16]. In contrast, the interaction between Rev-erbα and DBC1 is not mediated by the N-terminal domain of DBC1. Still, the N-terminal region of DBC1 is clearly important to regulate the stability and function of Rev-erbα.

DBC1 was also reported to modulate transcription activity of RARα (retinoic acid receptor α) [31], ERβ [14] and BRCA1 (breast cancer early-onset 1) [32], suggesting that DBC1 could be a more general regulator of transcription. Because DBC1 also regulates the deacetylases HDAC3 and SIRT1, it will be of interest to explore whether deacetylases are involved in the regulation of gene transcription by DBC1. For instance, DBC1 regulation of ERα involves inhibition of the SIRT1–ERα interaction and deacetylation of the receptor [16]. Other receptors have also been shown to be acetylated, such as the nuclear bile acid receptor (FXR) [33], AR [34] and LXR (liver X receptor) [35], and their deacetylation is regulated by SIRT1. However, SIRT1 can function either as a co-activator or a co-repressor for these receptors, implying that acetylation can activate or inhibit nuclear receptor function. Given that Rev-erbα interacts with the HDAC3 deacetylase, it will be interesting to investigate whether Rev-erbα is acetylated and whether deacetylation is a mechanism of regulation of this receptor. Furthermore, it will be important to determine whether the DBC1 effect on Rev-erbα requires HDAC3.

The overall picture emerging is that Rev-erbα, NCoR and HDAC3 co-ordinate the circadian regulation of liver fat metabolism and clock genes [25,36]. Loss of HDAC3 or Rev-erbα led to hepatosteatosis, although it is more pronounced in mice lacking HDAC3 than Rev-erbα [30,36]. However, recent studies show that mice that have both Rev-erbα and Rev-erbβ knocked out have a marked increase in hepatosteatosis and deregulation of several metabolic and clock genes [37,38]. These findings establish that both variants of Rev-erb work together to protect the organism from major alterations in circadian and metabolic processes. Because the DBC1-KO mice are protected against HFD-induced liver steatosis [18], it is important to determine the molecular pathways regulated by DBC1. Under normal diet conditions, DBC1-KO mice have lower levels of Rev-erbα, but have higher HDAC3 activity. However, we still do not know how these pathways are regulated under conditions of HFD and whether DBC1 also regulates Rev-erbβ. Elucidating the connections between DBC1, HDAC3 and Rev-erbs may have implications for the pathogenesis and treatment of metabolic diseases such as obesity, diabetes, liver steatosis and metabolic syndrome. The recent development of Rev-erb agonists that alter circadian behaviour, and decrease obesity and adipogenesis [22,23] suggests that these pathways can be targeted to improve circadian rhythm and metabolism. In this regard, it is possible that targeting the DBC1–Rev-erbα interaction may have important implications for the treatment of metabolic diseases.

Finally, DBC1 is required for the cellular circadian oscillations of Rev-erbα and BMAL1. This suggests that DBC1 could be an important regulator of circadian rhythms. Further studies will be necessary to determine whether DBC1-KO animals show alterations in circadian behaviours, such as changes in the circadian period length, or responses to 12 h light/12 h dark. On the basis of our observations, we propose that DBC1 is a novel regulator of both circadian and metabolic pathways.

## Online data

Supplementary data

## References

[1] Bellet M. M., Sassone-Corsi P. (2010). Mammalian circadian clock and metabolism: the epigenetic link. J. Cell Sci..

[2] Duez H., Staels B. (2009). Rev-erb-α: an integrator of circadian rhythms and metabolism. J. Appl. Physiol..

[3] Feng D., Lazar M. A. (2012). Clocks, metabolism, and epigenome. Cell.

[4] Yin L., Lazar M. A. (2005). The orphan nuclear Rev-erbα recruits the N-CoR/histone deacetylase 3
corepressor to regulate the circadian *Bmal1* gene. Mol. Endocrinol..

[5] Crumbley C., Burris T. P. (2011). Direct regulation of *CLOCK* expression by REV-ERB. PLoS ONE.

[6] Yin L., Wu N., Curtin J. C., Qatanani M., Szwergold N. R., Reid R. A., Waitt G. M, Parks D. J., Pearce K. H., Wisely B., Lazar M. A. (2007). Rev-erbα, a heme sensor that coordinates metabolic and circadian
pathways. Science.

[7] Wu N., Yin L., Hanniman E. A., Joshi S., Lazar M. A. (2010). Negative feedback maintenance of heme homeostasis by its receptor,
Rev-erbα. Genes Dev..

[8] Wang J., Lazar M. A. (2008). Bifunctional role of Rev-erbα in adipocyte differentiation. Mol. Cell. Biol..

[9] Guenther M. G., Barak O., Lazar M. A. (2001). The SMRT and N-CoR co-repressors are activating cofactors for histone deacetylase
3. Mol. Cell. Biol..

[10] Ishizuka T., Lazar M. A. (2003). The NCoR/histone deacetylase 3 complex is required for repression by thyroid hormone
receptor. Mol. Cell. Biol..

[11] Raghuram S., Stayrook K. R., Huang P., Rogers P. M., Nosie A. K., McClure D. B., Burris L. L., Khorasanizadeh S., Burris T. P., Rastinejad F. (2007). Identification of heme as the ligand for the orphan nuclear receptor Rev-erbα
and Rev-erbβ. Nat. Struct. Mol. Biol..

[12] Yin L., Wang J., Klein P. S., Lazar M. A. (2006). Nuclear receptor Rev-erbα is a critical lithium-sensitive component of the
circadian clock. Science.

[13] Fu J., Jiang J., Li J., Wang S., Shi G., Feng Q., White E., Qin J., Wong J. (2009). Deleted in breast cancer 1, a novel androgen receptor (AR) coactivator that promotes
AR DNA-binding activity. J. Biol. Chem..

[14] Koyama S., Wada-Hiraike O., Nakagawa S., Tanikawa M., Hiraike H., Miyamoto Y., Sone K., Oda K., Fukuhara H., Nakagawa K. (2010). Repression of estrogen receptor β function by putative tumor suppressor
DBC1. Biochem. Biophys. Res. Commun..

[15] Trauemicht A. M., Kim S. J., Kim N. H., Boyer T. G. (2008). Modulation of estrogen receptor α protein level and survival function by
DBC1. Mol. Endocrinol..

[16] Yu E. J., Seok-Hyung K., Heo K., Ou C.-Y., Stalicup M. R., Kim J. H. (2011). Reciprocal roles of DBC1 and SIRT1 in regulating estrogen receptor α activity
and co-activator synergy. Nucleic Acid Res..

[17] Chini C. C. S., Escande C., Nin V., Chini E. N. (2010). HDAC3 is negatively regulated by the nuclear protein DBC1. J. Biol. Chem..

[18] Escande C., Chini C. C., Nin V., Dykhouse K. M., Novak C. M., Levine J., van Deursen J., Gores G. J., Lou Z., Chini E. N. (2010). Deleted in breast cancer-1 regulates SIRT1 activity and contributes to high-fat
diet-induced liver steatosis in mice. J. Clin. Invest..

[19] Kim J. E., Chen J., Lou Z. (2008). DBC1 is a negative regulator of SIRT1. Nature.

[20] Zhao W., Kruse J.-P., Tang Y., Jung S. Y., Qin J., Gu W. (2008). Negative regulation of the deacetylase SIRT1 by DBC1. Nature.

[21] Gibbs J. E., Blaikley J., Beesley S., Matthews L., Simpson K. D., Boyce S. H., Farrow S. N., Else K. J., Singh D., Ray D. W., Loudon A. S. (2012). The nuclear receptor Rev-erbα mediates circadian regulation of innate immunity
through selective regulation of inflammatory cytokines. Proc. Natl. Acad. Sci. U.S.A..

[22] Kumar N., Solt L. A., Wang Y., Rogers P. M., Bhattacharyya G., Kamenecka T. M., Stayrook K. R., Crumbley C., Floyd E., Gimble J. M. (2010). Regulation of adipogenesis by natural and synthetic Rev-erb ligands. Endocrinology.

[23] Solt L. A., Wang Y., Baanerjee S., Hughes T., Kojetin D. J., Lundasen T., Shin Y., Liu J., Cameron M. D., Noel R. (2012). Regulation of circadian behavior and metabolism by synthetic Rev-erb
agonists. Nature.

[24] Yin L., Joshi S., Wu N., Tong X., Lazar M. A. (2010). E3 ligases Arf-bp1 and Pam mediate lithium-stimulated degradation of the circadian
heme receptor Rev-erbα. Proc. Natl. Acad. Sci. U.S.A..

[25] Yin L., Wu N., Lazar M. A. (2010). Nuclear receptor Rev-erbα: a heme receptor that coordinates circadian rhythm
and metabolism. Nucl. Recept. Signaling.

[26] Preitner N., Damiola F., Lopez-Molina L., Zakany J., Duboule D., Albrecht U., Schibler U. (2002). The orphan nuclear receptor Rev-erbα controls circadian transcription within
the positive limb of the mammalian circadian oscillator. Cell.

[27] Wang J., Yin L., Lazar M. A. (2006). The orphan nuclear receptor Rev-erbα regulates circadian expression of
plasminogen activator inhibitor type 1. J. Biol. Chem..

[28] Nakahata Y., Kaluzova M., Grimaldi B., Sahar S., Hirayama J., Chen D., Guarente L. P., Sassone-Corsi P. (2008). The NAD^+^-dependent deacetylase SIRT1 modulates CLOCK-mediated chromatin
remodeling and circadian control. Cell.

[29] Asher G., Gatfield D., Stratmann M., Reinke H., Dibner C., Kreppel F., Mostoslavsky R., Alt F. W., Schibler U. (2008). SIRT1 regulates circadian *clock* gene expression through PER2
deacetylation. Cell.

[30] Feng D., Liu T., Sun Z., Bugge A., Mullican S. E., Alenghat T., Liu X. S., Lazar M. A. (2011). A circadian rhythm orchestrated by histone deacetylase 3 controls hepatic liver
metabolism. Science.

[31] Garapaty S., Xu C. E., Trojer P., Mahajan M. A., Neubert T. A., Samuels H. H. (2009). Identification and characterization of a novel nuclear protein complex involved in
nuclear hormone receptor-mediated gene regulation. J. Biol. Chem..

[32] Hiraike H., Wada-Hiraike O., Nakagawa S., Koyama S., Miyamoto Y., Sone K., Tanikawa M., Tsuruga T., Nagasaka K., Matsumoto Y. (2010). Identification of DBC1 as a transcriptional repressor for BRCA1. Br. J. Cancer.

[33] Kamper J. K., Xiao Z., Ponugoti B., Miao J., Kanamaluru D., Tsang S., Wu S. Y., Chiang C. M., Veenstra T. D. (2009). FXR acetylation is normally dynamically regulated by p300 and SIRT1 but
constitutively elevated in metabolic disease states. Cell Metab..

[34] Dai Y., Ngo D., Forman L. W., Qin D. C., Jacob J., Faller D. V. (2007). Sirtuin 1 is required for antagonist-induced transcriptional repression of
androgen-responsive genes by the androgen receptor. Mol. Endocrinol..

[35] Lin X., Zhang S., Blander G., Tse J. G., Krieger M., Guarente L. (2007). SIRT1 deacetylates and positively regulates the nuclear receptor LXR. Mol. Cell.

[36] Alenghat T., Meyers K., Mullican S. E., Leitner K., Adeniji-Adele A., Avila J., Bucan M., Ahima R. S., Kaestner K. H., Lazar M. A. (2008). Nuclear receptor corepressor and histone deacetylase 3 govern circadian metabolic
physiology. Nature.

[37] Bugge A., Feng D., Everett E. R., Mullica S. E., Wang F., Jager J., Lazar M. A. (2012). Rev-erbα and Rev-erbβ coordinately protect the circadian clock and
metabolic function. Genes Dev..

[38] Cho H., Zhao X., Hatori M., Yu R. T., Barish G. D., Lam M. T., Chong L. W., Ditacchio L., Atkins A. R., Glass C. K. (2012). Regulation of circadian behavior and metabolism by Rev-erbα and
Rev-erbβ. Nature.

