# Supplementary material for: DBC1 (Deleted in Breast Cancer 1) modulates the stability and function of the nuclear receptor Rev-erbα
Source: Biochem J. 2013 Apr 12;451(Pt 3):453–61. doi: 10.1042/BJ20121085 (PMC3630992; doi:10.1042/BJ20121085)
Supplement: Supplementary data [file bj4510453add.pdf]

Biochem. J. (2013) **451**, 453–461 (Printed in Great Britain) doi:10.1042/BJ20121085

SUPPLEMENTARY ONLINE DATA

DBC1 (Deleted in Breast Cancer 1) modulates the stability and function of the nuclear receptor Rev-erbα

Claudia C. S. CHINI, Carlos ESCANDE, Veronica NIN and Eduardo N. CHINI[1]

Laboratory of Signal Transduction, Department of Anesthesiology, Mayo Clinic College of Medicine, Rochester, MN 55905, U.S.A.

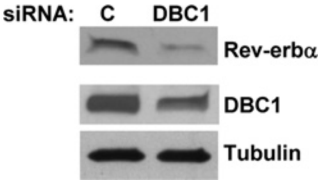

Figure S1 INS-1 cells were treated with control and DBC1 siRNAs

Cell lysates were immunoblotted with anti-Rev-erbα, anti-DBC1 and anti-tubulin antibodies.

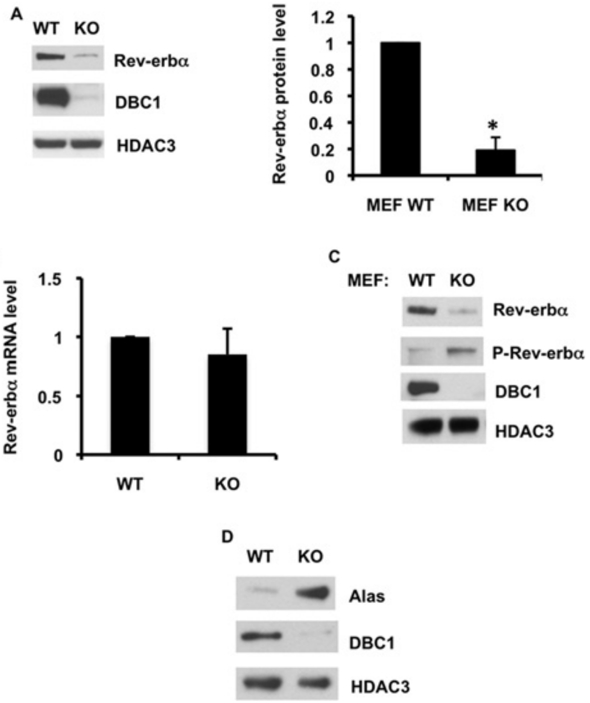

Figure S2 Rev-erbα and Alas levels in DBC1 WT and DBC1-KO MEFs

(**A**) Cell lysates from DBC1 WT and DBC1-KO MEFs were immunoblotted with anti-Rev-erbα, anti-DBC1 and anti-HDAC3 antibodies. The histogram shows the means ± S.D. for five independent experiments. HDAC3 was used as a loading control, since we have shown previously that HDAC3 protein levels are not regulated by DBC1 [1]. (**B**) Rev-erbα mRNA levels were measured in DBC1 WT and DBC1-KO MEFs. mRNA levels were quantified by real-time PCR. The histogram shows the means ± S.D. ($n = 3$). (**C**) DBC1 WT and DBC1-KO MEFs were immunoblotted with anti-Rev-erbα, anti-phospho-Rev-erbα, anti-DBC1 and anti-HDAC3 antibodies. (**D**) Cell lysates from DBC1 WT and DBC1-KO MEFs were immunoblotted with anti-Alas, anti-DBC1 and anti-HDAC3 antibodies.

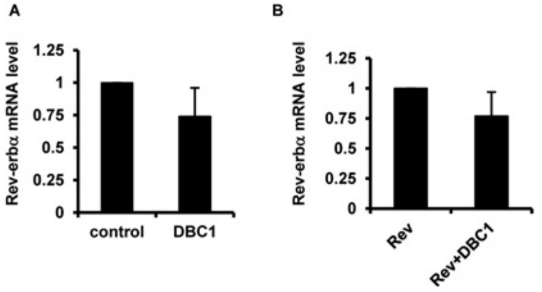

Figure S3 Rev-erbα and mRNA levels in HEK-293T cells

HEK-293T cells were transfected with vector and DBC1 (**A**) or Rev-erbα in the presence of vector or DBC1 (**B**). Rev-erbα mRNA levels were quantified by real-time PCR. The histograms show the means ± S.D. for three experiments.

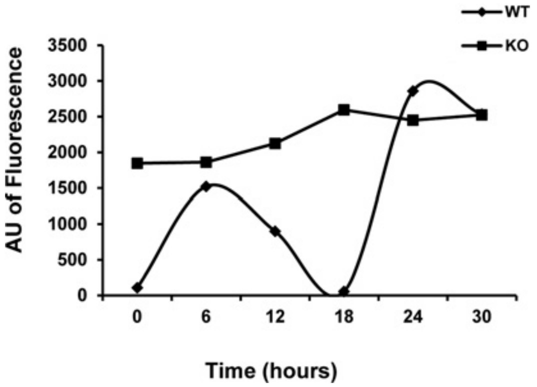

Figure S4 SIRT1 activity in DBC1 WT and DBC1-KO MEFs after serum shock

MEFs from WT and DBC1-KO mice were starved, serum shocked and released in the starvation medium. The times indicate the number of hours after serum shock. At the indicated times, cells were collected and SIRT1 activity was measured as described previously [2].

REFERENCES

1 Chini, C. C. S., Escande, C., Nin, V. and Chini, E. N. (2010) HDAC3 is negatively regulated by the nuclear protein DBC1. J. Biol. Chem. **285**, 40830–40837

2 Escande, C., Chini, C. C., Nin, V., Dykhouse, K. M., Novak, C. M., Levine, J., van Deursen, J., Gores, G. J., Lou, Z. and Chini, E. N. (2010) Deleted in breast cancer-1 regulates SIRT1 activity and contributes to high-fat diet-induced liver steatosis in mice. J. Clin. Invest. **120**, 545–558

Received 6 July 2012/15 January 2013; accepted 12 February 2013
Published as BJ Immediate Publication 12 February 2013, doi:10.1042/BJ20121085

[1] To whom correspondence should be addressed (email chini.eduardo@mayo.edu).

© 2013 The Author(s)
The author(s) has paid for this article to be freely available under the terms of the Creative Commons Attribution Licence (CC-BY) (http://creativecommons.org/licenses/by/3.0/) which permits unrestricted use, distribution and reproduction in any medium, provided the original work is properly cited.

Biochemical Journal www.biochemj.org
